# Modeling the daily rhythm of human pain processing in the dorsal horn

**DOI:** 10.1371/journal.pcbi.1007106

**Published:** 2019-07-11

**Authors:** Jennifer Crodelle, Sofia H. Piltz, Megan Hastings Hagenauer, Victoria Booth

**Affiliations:** 1 Courant Institute of Mathematical Sciences, New York University, New York, New York, United States of America; 2 Department of Mathematics, University of Michigan, Ann Arbor, Michigan, United States of America; 3 Molecular and Behavioral Neuroscience Institute, University of Michigan, Ann Arbor, Michigan, United States of America; New Jersey Institute of Technology, UNITED STATES

## Abstract

Experimental studies show that human pain sensitivity varies across the 24-hour day, with the lowest sensitivity usually occurring during the afternoon. Patients suffering from neuropathic pain, or nerve damage, experience an inversion in the daily modulation of pain sensitivity, with the highest sensitivity usually occurring during the early afternoon. Processing of painful stimulation occurs in the dorsal horn (DH), an area of the spinal cord that receives input from peripheral tissues via several types of primary afferent nerve fibers. The DH circuit is composed of different populations of neurons, including excitatory and inhibitory interneurons, and projection neurons, which constitute the majority of the output from the DH to the brain. In this work, we develop a mathematical model of the dorsal horn neural circuit to investigate mechanisms for the daily modulation of pain sensitivity. The model describes average firing rates of excitatory and inhibitory interneuron populations and projection neurons, whose activity is directly correlated with experienced pain. Response in afferent fibers to peripheral stimulation is simulated by a Poisson process generating nerve fiber spike trains at variable firing rates. Model parameters for fiber response to stimulation and the excitability properties of neuronal populations are constrained by experimental results found in the literature, leading to qualitative agreement between modeled responses to pain and experimental observations. We validate our model by reproducing the wind-up of pain response to repeated stimulation. We apply the model to investigate daily modulatory effects on pain inhibition, in which response to painful stimuli is reduced by subsequent non-painful stimuli. Finally, we use the model to propose a mechanism for the observed inversion of the daily rhythmicity of pain sensation under neuropathic pain conditions. Underlying mechanisms for the shift in rhythmicity have not been identified experimentally, but our model results predict that experimentally-observed dysregulation of inhibition within the DH neural circuit may be responsible. The model provides an accessible, biophysical framework that will be valuable for experimental and clinical investigations of diverse physiological processes modulating pain processing in humans.

## Author summary

Human pain sensitivity follows a daily (∼24 hour) rhythm. In particular, humans experience the highest sensitivity to pain in the middle of night and lowest in the afternoon. Patients suffering from neuropathy, a disease resulting from nerve damage leading to an increase in pain sensitivity, experience an approximately 12-hour shift in their rhythmicity such that the highest sensitivity occurs in the afternoon. Neuropathy is a difficult condition to treat since it is often unfeasible to locate the damaged nerve and it is also unclear how this damage causes a shift in rhythmicity and an increase in pain. Understanding the mechanism underlying the shift in rhythmicity may lead to improvements in the knowledge of the transmission of pain from the damaged nerve to the pain-processing center in the spinal cord, and thus better treatment protocols. We have built a population-based model to describe this transmission with a particular focus on daily rhythms. We show that our model reproduces experimentally-observed rhythmicity of both normal pain responses, as well as neuropathic pain. Our model predicts that a potential mechanism underlying the shift in rhythmicity for neuropathic pain is a change in the interaction of the nerve fibers from inhibition to excitation.

## Introduction

The processing of pain engages a wide variety of neural circuits across the nervous system including those in the spinal cord, brainstem, thalamus, and cortex. More specifically, it is thought that the dorsal horn (DH), an area of the spinal cord, serves as the initial processing center for incoming nociceptive, or painful signals, with the midbrain and cortex providing top-down modulation to that circuitry [1]. As a result, there is a tradition of modeling pain processing by focusing exclusively on spinal cord circuitry. This circuitry receives information about stimulation of peripheral tissues from several types of primary afferent nerve fibers. These afferents have their cell bodies in the dorsal root ganglia (DRG), a cluster of nerve cell bodies located exterior to the spinal cord, and their axons (or fibers) target the DH [2]. Responses to innocuous stimulation are carried by rapidly conducting A*β*-fibers [3], whereas nociceptors (i.e., nerve fibers that detect painful stimuli) are only activated when a stimulus exceeds a specific threshold. There are two major classes of nociceptive fibers: fast conducting A*δ*-fibers that mediate localized, fast pain and small-diameter C-fibers that mediate diffused, slow pain. Among the neuronal populations in the DH, the projection neurons (PNs) receive input from all fibers and constitute the majority of the output from the dorsal horn circuit up to the brain.

In this article, we introduce a biophysically-based, mathematical model of the nociception-processing neural circuit in the DH, which expands on our earlier work [4]. We are particularly interested in using the model to investigate mechanisms for daily (i.e., diurnal) modulation of pain sensitivity. In many clinical conditions, pain sensitivity follows a daily cycle [5], that is, it exhibits a trough in the late afternoon and a peak sometime after midnight for humans [6], but it is currently unclear how much of that rhythmicity is derived from daily fluctuation in the underlying causes of the pain versus rhythmicity in the neural processing of pain. Within the experimental pain literature, rhythmic influences on pain sensation occur regardless of whether pain responses are measured subjectively or objectively [7–10], suggesting that the rhythmic modulation of pain responses occurs at the level of basic nociceptive processing. This rhythmic modulation of pain sensitivity also increases with pain intensity [9, 11, 12]. Furthermore, rhythmic influences on pain sensitivity are detectable in experiments involving a variety of different kinds of nociceptive stimuli, including cold, heat, electric current, pressure, and ischemia (see Tables 1–2 in [6]). Interestingly, experimental studies have also shown daily rhythmicity in tactile discrimination in nearly opposite phase to pain sensitivity, namely highest tactile sensitivity occurring in the late afternoon and lowest in the morning [13].

There are several hypotheses for the source of the daily rhythm in pain sensitivity, including central nervous system, spinal, and peripheral mechanisms [5, 14–18]. Recent studies show that cells in the DRG rhythmically express the primary genes responsible for generating an intrinsic 24-hour, or circadian, rhythmicity of other physiological processes, including Bmal1, Clock, *Per1* and *Per2* [15, 16]. In addition, the rhythm in behavioral nociception followed the gene expression rhythm [15] and disruption of their expression affected behavioral pain responses [16]. These findings motivate our use of a spinal cord model to test questions regarding daily influences on pain processing. As such, the model assumes that the daily modulation occurs at the level of primary afferent input to the spinal cord circuitry. Additionally, we specifically model the portion of experienced pain that arises from nociceptive input to the spinal cord and ignore any potential sources of top-down modulation.

As concerns the connections between neuron populations in the DH, there are several proposed circuitries for the processing of touch, nociception, and itch (see, e.g., [19, 20]). In this work, we take an approach similar to previous models of spinal cord nociception processing (e.g., [21]) and employ the network architecture in the DH proposed by the gate control theory of pain [22]. In doing so (and when we introduce daily modulation), we note that the aim of our work here is focused on the processing of painful, noxious stimulation, not mechanical, non-noxious stimulation, which we acknowledge may have a different circuitry (for a review of circuitries for mechanical pain and itch, see [23]).

The gate control theory of pain [22, 24] posits that the neural circuitry in the DH exhibits a gating mechanism that is modulated by activity in the A*β*- and C-fibers [25]. Specifically, nociceptive C-fiber-facilitated activity in the DH circuit is inhibited by A*β*-fiber activity. When the amount of painful stimuli (i.e., activity in the C-fibers) outweighs the inhibition from the A*β*-fibers, the “gate opens” and activates the PNs (and the experience of pain). Although the gate control theory of pain [22] is a simplification and not a complete representation of the physiological underpinnings of pain processing [25], it has been a productive starting point for several mathematical and computational models of pain [21, 26–28].

For our model of the DH circuit, we implement a neuronal population firing-rate model formalism [29, 30] to describe the population activity of projection, inhibitory, and excitatory neurons in the DH. Our choice of this commonly-used model formalism is based on the large number of afferent fibers and neurons in the DH, and the assumption that the majority of information flow in the DH circuit is through firing rates of neural populations rather than in specific spike timing within the populations [30, 31]. An advantage of this formalism is its biophysical basis and relative simplicity, thus making our model an accessible theoretical framework for experimental and clinical investigations of diverse physiological processes modulating pain processing in humans.

The rest of the paper is organized as follows. In the Methods section, we formulate the equation system of the neural circuit for pain processing in the DH, describing the time evolution of the average firing rates of the excitatory and inhibitory interneuron, and PN populations in response to input on the afferent nerve fibers. The model includes NMDA-mediated synaptic input from the C-fibers to the PNs that depends on postsynaptic activity. We also describe the use of a Poisson process to simulate neural spikes on the afferent fibers that represent the input from the DRG to the DH and the interactions between the afferent fibers incorporated in our model, respectively. In the Results section, we present validation studies for our model including reproduction of the wind-up phenomenon.

With our principal aim to investigate the daily rhythmicity of pain sensation, we apply the model to predict the daily modulation of the pain inhibition phenomena. As a novel application of the model, we investigate effects of experimentally-observed dysregulation of inhibition within the DH circuit under neuropathic pain conditions (i.e., a chronic condition with persistent pain experience associated, e.g., with peripheral nerve damage) on the daily modulation of pain sensitivity. We find that dysregulation of A*β*-fiber dependent presynaptic inhibition of C-fiber signaling can account for it. Finally, we discuss limitations and future modifications, as well as importance and application, of our model in the Discussion.

## Methods

### Model equations

**Fig 1:**
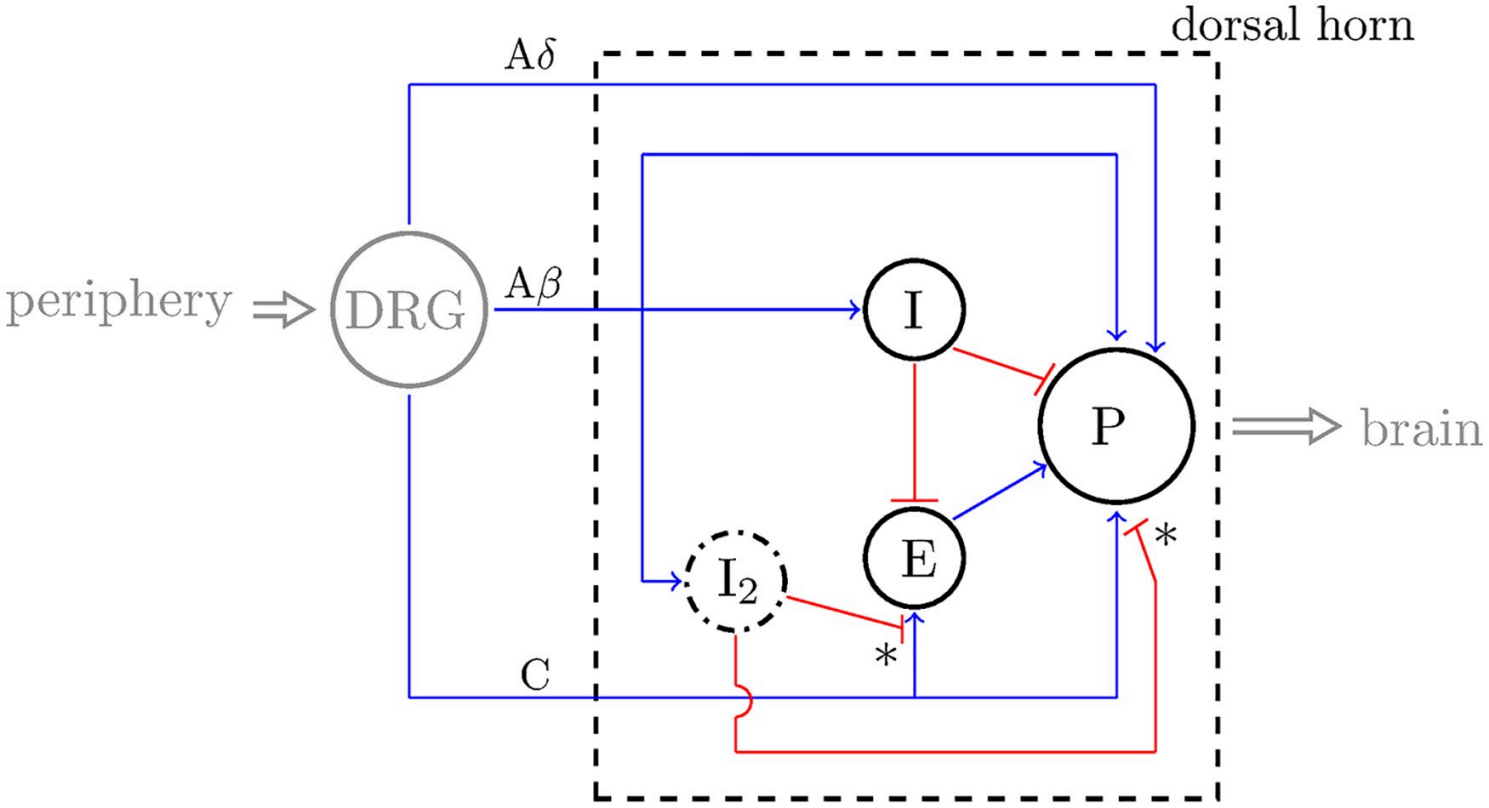
Diagram of our model of the dorsal horn (DH) circuit (within the dashed rectangle) including connections between the neuron populations I, E, and P, the afferent fibers A*β*, A*δ*, and C, and the dorsal root ganglion (DRG). We denote inhibitory connections with bars (in red) and excitatory connections with arrows (in blue). The dash-dotted line represents an inhibitory interneuron population (I_2_) that is modeled indirectly.

We construct a model describing the spinal processing of nociceptive stimuli in humans by considering the average firing rate of three populations of neurons in the DH: the PNs (P), inhibitory (I) interneurons, and excitatory (E) interneurons, in response to the average firing rate of the A*β*-, A*δ*-, and C-afferent fibers (see Fig 1). In this work, we expand on a model developed in [4], which follows the modeling approach similar to [26] with the exception that our model predictions are in terms of average firing rates of neuron populations [29] instead of average membrane potentials. In contrast to our previous model in [4], the new elements of the model introduced in this work consist of including i) Poisson processes to generate spiking activity on the input nerve fibers, ii) NMDA receptor-mediated synaptic interactions and iii) an additional inhibitory interneuron population I_2_, and iv) removal of the connection to the midbrain. These four modifications allow us to i) represent a biologically realistic fiber input to which the model is robust, ii) reproduce experimentally observed frequency effects during wind-up, iii) expand the model parameter range that replicates patterns seen in experiments on neuropathy, and vi) focus solely on modeling spinal-cord processing of pain, respectively.

As concerns the general structure of connections between the neuronal populations in the circuit (dashed rectangle in Fig 1), we follow previous models of pain and use the circuitry presented, e.g., in [21]. Briefly, PNs receive direct synaptic input from the three afferent fiber types, A*β*-fibers excite inhibitory interneurons and C-fibers excite excitatory interneurons. Both interneuron populations synapse onto the PNs and the inhibitory interneurons inhibit the excitatory interneurons. We also include A*β*-dependent presynaptic inhibition of C-fiber activity mediated through an additional inhibitory interneuron population (I_2_) that is modeled indirectly [see Eq (5)]. We assume that the input to our model circuit is a stimulation of the afferent fibers that has been pre-processed in the DRG. Based on fiber input and the connections between the neuron populations in the DH, our model computes the activity of the PNs, (P in Fig 1), whose output directly corresponds to the amount of pain experienced [32]. We note that there are many nuances in the perception of pain, including those originating in the cortex; however, we model the portion of pain that stems from nociceptive input to the spinal cord since it has been shown that pain perception correlates strongly with the firing rate of the PNs in the spinal cord [32, 33].

**Fig 2:**
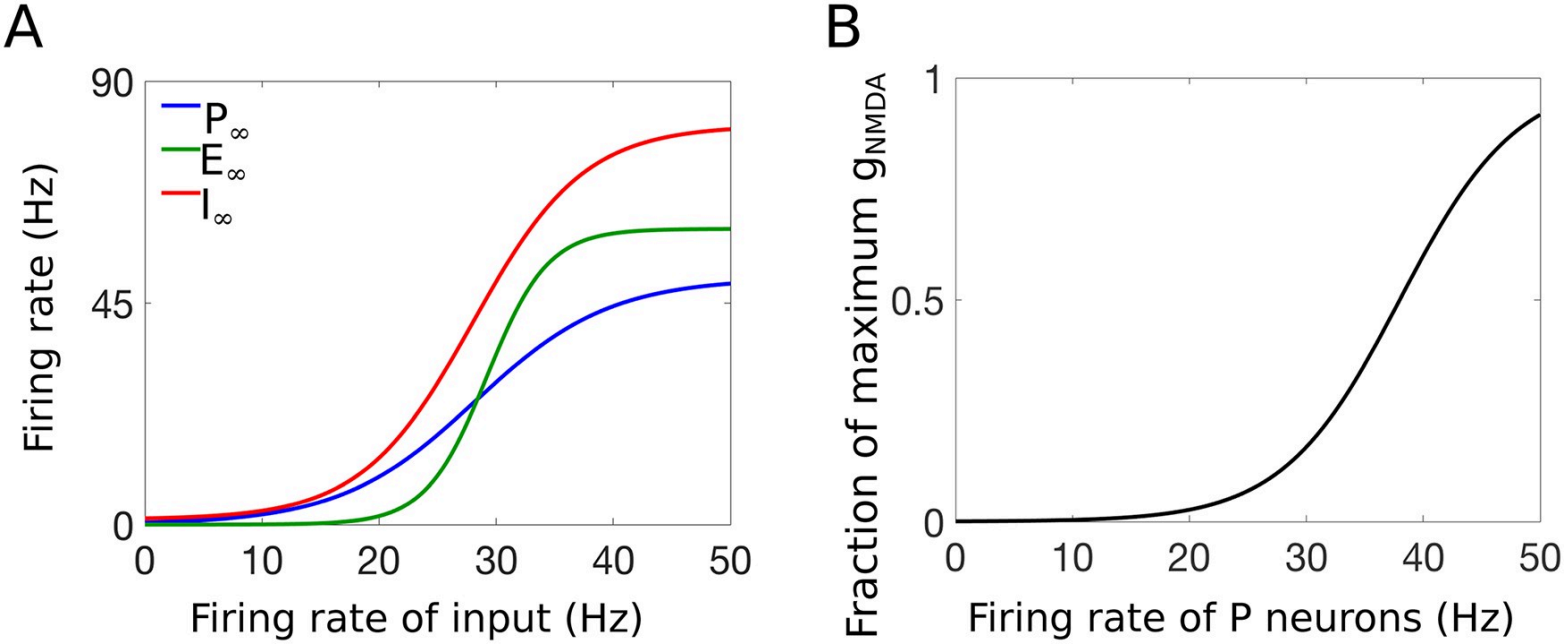
Response curve and NMDA activation plots [see Eq (3)]. A: Response functions of the projection (blue), excitatory (green), and inhibitory (red) neural populations for varying average input firing rates (on the x-axis). Parameters for these curves were chosen to match experiments presented in [21]. B: Activation curve for the NMDA-mediated synaptic input (M_∞_), shown as a fraction of the maximum synaptic weight *g*_NMDA_.

According to the formalism of firing-rate models, e.g., [29], we assume that the rate of change of the average firing rate in spikes per second (Hz) of the projection, inhibitory, and excitatory neuron populations, *f*_P_, *f*_I_, and *f*_E_, respectively, is determined by a nonlinear response function (see Fig 2A). These response functions determine the average firing rate response of a neuron population to a combination of external inputs (i.e., stimulations of the afferent fibers pre-processed in the DRG) and the firing rates of the presynaptic neuron populations (see Fig 1). In the absence of input from other neuron populations and afferent fibers, the average firing rate of the neuron population decays exponentially. These assumptions yield the following set of equations for the average firing rate of each population:

$$\begin{array}{c} \frac{df_{\text{P}}}{dt}=\frac{{\text{P}}_{\infty }\left(g_{\text{A}\beta \text{P}}f_{\text{A}\beta }\left(t\right)+g_{\text{A}\delta \text{P}}f_{\text{A}\delta }\left(t\right)+\left(g_{\text{CP}}+g_{\text{NMDA}}\right)f_{\text{C}}\left(t\right)+g_{\text{EP}}f_{\text{E}}-g_{\text{IP}}f_{\text{I}}\right)-f_{\text{P}}}{\tau_{\text{P}}}\;,\\ \frac{df_{\text{E}}}{dt}=\frac{{\text{E}}_{\infty }\left(g_{\text{CE}}f_{\text{C}}\left(t\right)-g_{\text{IE}}f_{\text{I}}\right)-f_{\text{E}}}{\tau_{\text{E}}}\;,\\ \frac{df_{\text{I}}}{dt}=\frac{{\text{I}}_{\infty }\left[g_{\text{A}\beta \text{I}}f_{\text{A}\beta }\left(t\right)\right]-f_{\text{I}}}{\tau_{I}}\;, \end{array}$$ (1)

where *t* is time in seconds, *τ*_P_ = 0.001 s, *τ*_E_ = 0.01 s, and *τ*_I_ = 0.02 s are the intrinsic time scales of the projection, excitatory, and inhibitory neuron populations, respectively. Weights *g*_*ij*_ denote the strength of the external input or connections from presynaptic neuron populations *i* (*i* = A*β*, A*δ*, C, P, E, I) to neuron population *j* (*j* = P, E, I). We indicate inhibitory synaptic input with a negative sign and excitatory synaptic input with a positive sign. We define the functions (of time *t*) for the external inputs, *f*_A*β*_(*t*), *f*_A*δ*_(*t*), and *f*_C_(*t*) in the next section.

The model includes N-methyl-D-aspartate (NMDA) type synapses from the C-fibers to the P population in the following way: to represent postsynaptic voltage-dependent removal of the magnesium (Mg^+^) block on NMDA receptors, we assume that the synaptic weight, *g*_NMDA_, depends on the average firing rate of the P population (*f*_P_), and thus, we consider *g*_NMDA_ as a variable that changes as a function of time (see Fig 2B), similar to [34]:

$$\begin{array}{c} \frac{dg_{\text{NMDA}}}{dt}=\frac{{\text{M}}_{\infty }\left(f_{\text{P}}\right)-g_{\text{NMDA}}}{\tau_{\text{NMDA}}}\;, \end{array}$$ (2)

where *τ*_NMDA_ = 1 s is the intrinsic time scale of the synaptic weight, *g*_NMDA_.

We assume a sigmoidal shape for the monotonically increasing firing rate response functions of the neuronal populations P_∞_, E_∞_, I_∞_, and the synaptic weight response function M_∞_, and use hyperbolic tangent functions to represent them as follows:

$$\begin{array}{cc} {\text{P}}_{\infty }\left(x\right) & ={\text{max}}_{\text{P}}\frac{1}{2}[1+\text{tanh}(\frac{1}{\alpha_{\text{P}}}(x-\beta_{\text{P}}))]\;,\\ {\text{E}}_{\infty }\left(x\right) & ={\text{max}}_{\text{E}}\frac{1}{2}[1+\text{tanh}(\frac{1}{\alpha_{\text{E}}}(x-\beta_{\text{E}}))]\;,\\ {\text{I}}_{\infty }\left(x\right) & ={\text{max}}_{\text{I}}\frac{1}{2}[1+\text{tanh}(\frac{1}{\alpha_{\text{I}}}(x-\beta_{\text{I}}))]\;,\\ {\text{M}}_{\infty }\left(x\right) & ={\text{max}}_{\text{M}}\frac{1}{2}[1+\text{tanh}(\frac{1}{\alpha_{\text{M}}}(x-\beta_{\text{M}}))]\;, \end{array}$$ (3)

where max_P_, max_E_, max_I_, and max_M_ are the maximum firing rates of the projection, excitatory, and inhibitory populations, and the maximum synaptic strength of the NMDA-mediated input, respectively. In Eq (3), the shape of the response functions is determined by the input *x* at which the average firing rate of the projection, excitatory, and inhibitory neuron population reaches half of its maximum value, *x* = *β*_P_, *x* = *β*_E_, and *x* = *β*_I_, respectively (see Fig 2A). The slope of the transition from non-firing to firing in the projection, excitatory, and inhibitory neuron population is given by 1/*α*_P_, 1/*α*_E_, and 1/*α*_I_, respectively. The activation of the NMDA synapse, M_∞_(*f*_P_), is modeled as an increasing function of the firing rate of the projection neurons, representing the resulting increase in synaptic strength as postsynaptic membrane potentials depolarize and the magnesium block of the NMDA receptors is released [34].

We choose parameter values for the response functions in such a way that the input-output curve of the projection, excitatory, and inhibitory neuron populations agrees qualitatively with experimental observations. Hence, we assume the inhibitory interneuron population has a nonzero resting firing rate, as has been reported in [1, 2], and a higher maximum firing rate than that of the projection and excitatory interneuron populations, as has been assumed in a biophysically detailed model of the DH circuit [21]. In our model assumptions for the response functions, we mimic the model predictions of [21] that agree with data from experimental observations in [35, 36].

As concerns the NMDA activation, we assume a similar sigmoidal shape but with a very slow rise time modeling the slow removal of magnesium ions from blocking the NMDA receptors with increase in cell activity. As the magnesium blockage is removed, the NMDA channels are clear to be activated and further depolarize the cell, resulting in an increase in the firing rate of the PNs. The function M_∞_(*f*_P_) models this activation of the NMDA channels resulting from the removal of the magnesium ions (see Fig 2B). All values of the parameters discussed above that we use in the numerical simulations of our model are listed in S1 Table.

### Model input from the DRG

To model input from the DRG, we simulate 1000 afferent fibers of three types that project to the DH. We note that our choice of 1000 fibers is based on the number of afferent fibers experimentally observed in one nerve bundle that projects to a skeletal muscle in the rat [37], which is on the order of 1000 [37, 38]. These three afferent fiber types differ not only in diameter sizes but also in the level of myelination. As a result, impulses are transmitted at different speeds in the three fiber types. The majority (82%) of these fibers are slow C-fibers (with an average conduction velocity of 0.5-2 m/s), 9% are A*δ*-fibers (with an average conduction velocity of 5-30 m/s), and 9% are A*β*-fibers (with an average conduction velocity of 30-70 m/s) [3, 38]. We assume that the times of initiation of activity in each of these fibers in response to nociceptive stimulation are roughly equivalent, resulting in the distribution of arrival times to the DH that has been experimentally observed, e.g., in Fig 1 of [39].

**Fig 3:**
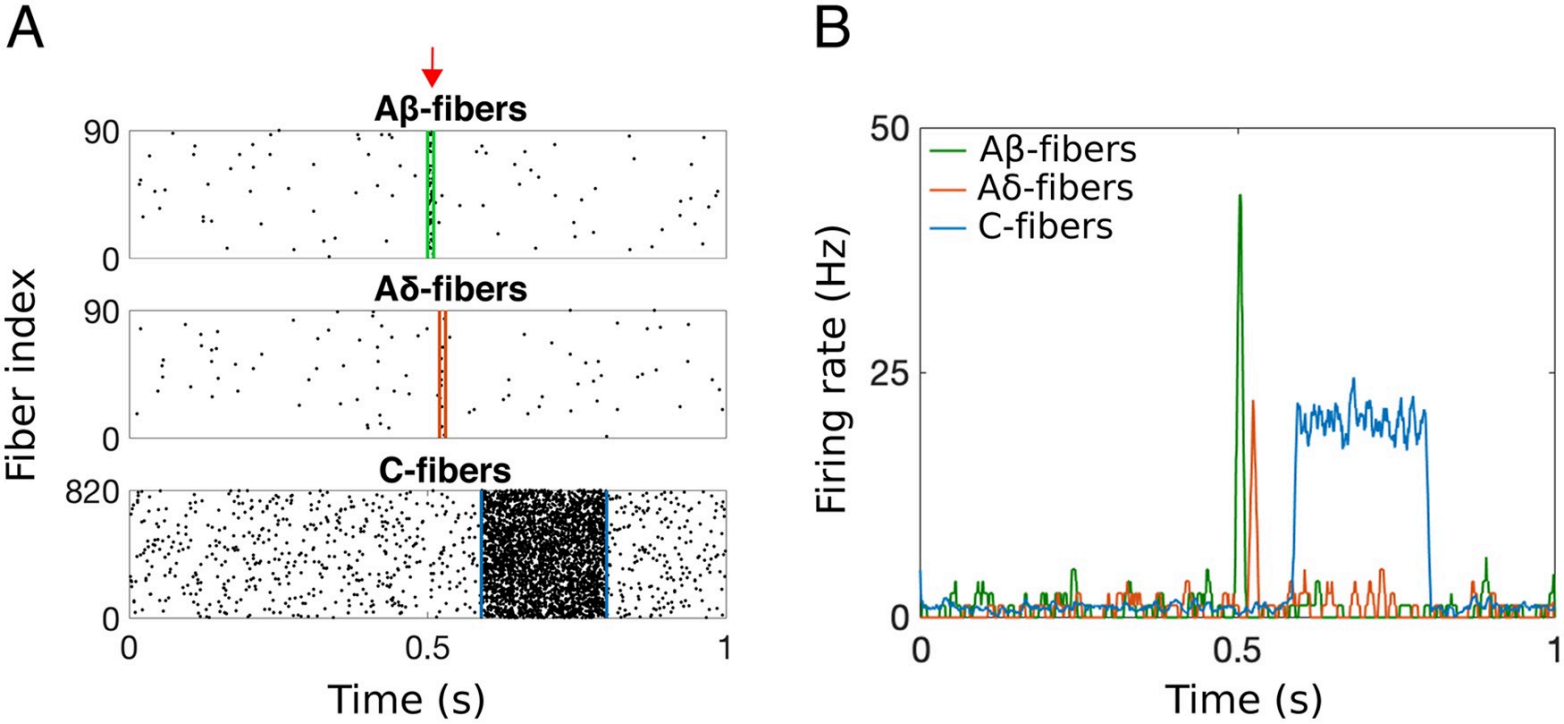
Simulated response of the populations of afferent nerve fibers to a brief nociceptive stimulus at *t* = 0.5 s (red arrow). A: Raster plots of spiking activity of (top) 90 A*β*-, (middle) 90 A*δ*-, and (bottom) 820 C-fibers with differing conductance speeds. B: The smoothed instantaneous firing rate (i.e., *f*_A*β*_(*t*), *f*_A*δ*_(*t*), and *f*_C_(*t*)) for each fiber population.

We aim to model nerve fiber activity from a brief nociceptive stimulus at the periphery (see Fig 1a in [39]). To do this, we use a Poisson process to simulate spike trains in the afferent fibers at a given firing rate. The activity of the afferent fibers in response to a brief nociceptive stimulus at t = 0.5 s can be seen in the raster plots in Fig 3A, where each small bar represents one spike/action potential and each row represents the activity in one afferent fiber over the course of 1 second. We consider the activity in 90 A*β*-, 90 A*δ*- and 820 C-fibers with baseline frequency of 1 Hz and stimulus response frequency of 40, 20, and 20 Hz, respectively. Each fiber has an increased firing rate for a set amount of time (10 ms for both A*β*- and A*δ*-fibers and 210 ms for C-fibers) chosen to replicate the response in the PNs as measured experimentally in [39]. We choose these increased firing rates for the afferent fibers to simulate a response to a nociceptive stimulus (see [33] and [40] for spiking dynamics of afferent fibers in response to varying levels of nociceptive stimuli) and a low background drive to simulate spontaneous activity of the fibers [41].

To compute the average firing rate in each of the three fiber groups, we compute an instantaneous firing rate by counting the number of spikes in a one-millisecond window of time, and then use a moving average with a time window of 10 ms to create a smooth firing rate function. As a result, our simulated input to the spinal cord on fibers with different conductance speeds reproduces the observed pattern [39] of fast, brief A*β*- and A*δ*-fiber activity (i.e., first pain) followed by delayed, longer lasting C-fiber activity (i.e., second pain). When simulating our model, we use these smoothed average firing rates (see Fig 3B) representing the response in the three fiber groups to a brief nociceptive stimulus as input to the DH circuit model.

### Daily modulation of input

**Fig 4:**
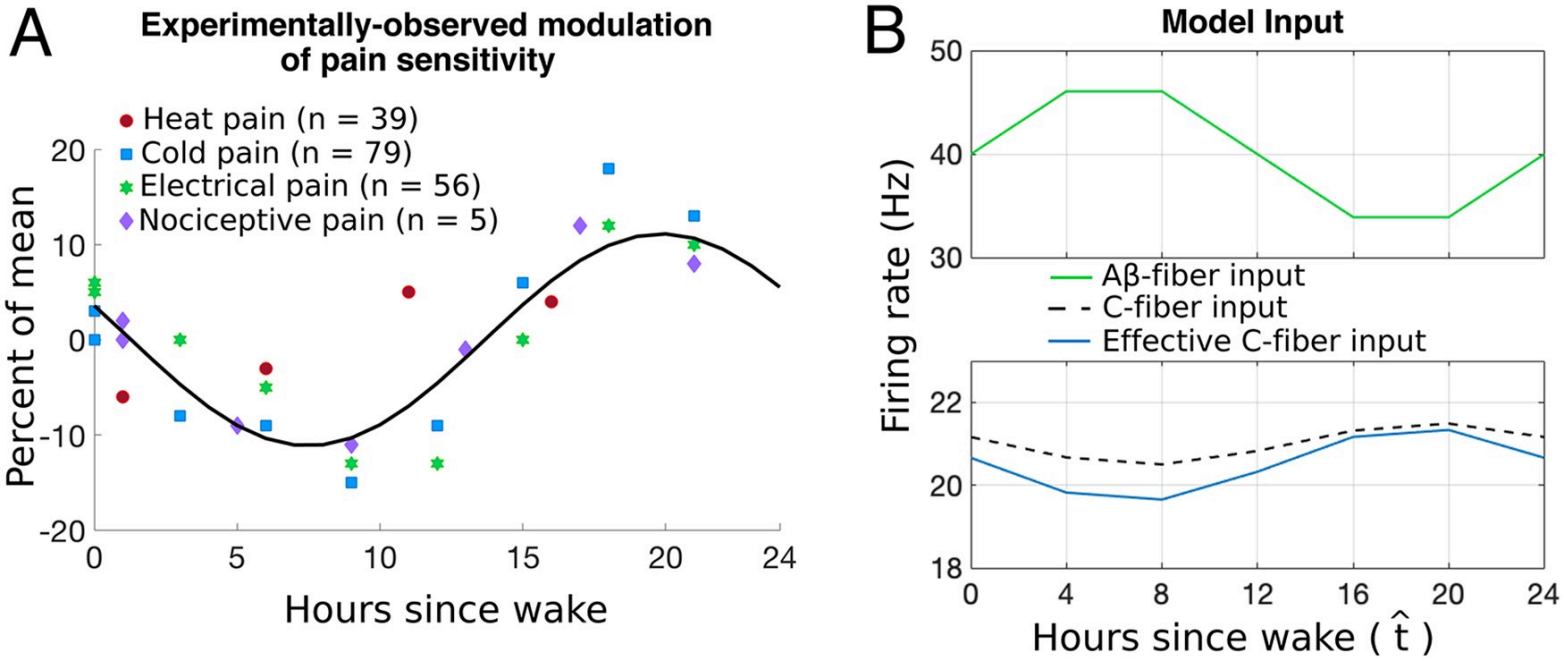
Daily rhythm in the modulation of pain sensitivity: Experiments and model. A: Prototypical human “daily pain sensitivity” (i.e., daily changes in pain sensitivity relative to mean pain sensitivity) function (*f*(*x*) = 11sin(0.25*x* + 2.8), where *x* ∈ [0, 24] hours) fitted to (symbols) data (*R*^2^ = 0.73 and RMSE = 4.69) from four experimental studies of pain responses. For more details and sources of these data, see [6]. B: Daily modulation of the stimulus-induced firing rate of the afferent fibers modeled by Eqs (4) and (5). Top(bottom) panel displays the daily modulation of the peak stimulus-induced firing rate of the A*β*- (C-) fibers. In the bottom panel, the blue curve represents the effective modulation of the C-fibers including A*β*-dependent presynaptic inhibition. The x-axis refers to hours since the typical or scheduled morning wake time.

Pain sensitivity follows a daily cycle in many clinical conditions [5]. There is strong evidence supporting rhythmicity in response to acute nociceptive stimuli [8, 11–13, 42]. In experiments where a rhythm in pain sensitivity was detected, its pattern is remarkably consistent, with pain sensitivity peaking during the hours when there is no daylight (and when humans are typically asleep), that is, from midnight to 5 AM [5]. In previous work, we analyzed experimental data reporting on the daily rhythm in human pain sensitivity from four studies investigating: 1) the threshold for forearm pain in response to heat (n = 39, [40]), 2) the threshold for tooth pain in response to cold (n = 79, [13]), 3) the threshold for tooth pain in response to electrical stimulation (n = 56, [13]), and 4) the threshold for nociceptive pain in response to electrical current (n = 5, 8]). The data points from these studies are shown in Fig 4A and details on the derivation of these data points can be found in [6]. We note here that we aligned the data to the subject’s typical or scheduled wake time (i.e., 0 hours after wake) and thus, clearly, this data represents a daily rhythm in pain sensitivity that includes sleep-wake-cycle effects that cannot be uncoupled from an endogenous circadian rhythm. The result is that, in this work, we discuss pain sensitivity as a function of hours since morning wake time to align our results with these data sources.

The data strongly suggest a sinusoidal profile, and thus we fit a sinusoidal function to the data using Matlab’s [43] curve fitting scheme (cftool) (see solid curve in Fig 4A). We hypothesize that this best-fit sinusoid (*R*^2^ = 0.73 and root mean-square error of 4.69) represents a prototypical daily rhythm in pain sensitivity for humans, with a sharp peak in pain sensitivity occurring close to midnight (following 18 hours of waking), and that then decreases during the night to reach a minimum in pain sensitivity in the afternoon (following 9 hours of wake, or approximately 4pm).

Experimental work also suggests a daily rhythm in the sensitivity of touch (see Figs 1 and 2 in [13]) with the highest sensitivity for tactile discrimination occurring in the late afternoon and the lowest sensitivity in the late morning [13]. Since cells in the DRG (that contains the cell bodies of the afferent fibers) rhythmically express clock genes responsible for generating rhythmicity of other physiological processes [15], we assume in our model that daily modulation occurs at the level of primary afferent input to the spinal cord. Furthermore, these experimental observations motivate us to introduce rhythmicity in the model input from *Aβ*-fibers that exhibits nearly a 12-hr shift from the rhythm of the C-fiber-model inputs. We note here that although we consider rhythmicity in the A*β*-fibers [13], our modeling work focuses on describing processing of nociceptive stimuli. Thus, our model does not simulate processing of strictly mechanical stimuli which may use different circuitry from that of nociceptive stimuli.

We use the sinusoidal curve obtained from fitting the experimental data in Fig 4A, with the slight modification of making the period exactly 24 hours, to modulate the A*β*- and C-fiber activity as a function of the time of day in hours since typical morning wake time. We implement daily rhythmicity in the firing rates of the A*β*- and C-fibers by varying their stimulation response frequencies, ${\text{R}}_{A\beta }\left(\hat{t}\right)$ and ${\text{R}}_{\text{C}}\left(\hat{t}\right)$, respectively, with approximately opposite phases. The average firing rates of the fibers (40 Hz for A*β*- fibers and 21 Hz for C-fibers) were estimated from experiments of receptor activity in the human hand [40]. This yields equations for the firing rates of the fibers over the day as follows:

$$\begin{array}{cc} {\text{R}}_{\text{A}\beta }\left(\hat{t}\right) & =6\sin(\frac{\pi }{12}\hat{t})+40\;,\\ {\text{R}}_{\text{C}}\left(\hat{t}\right) & =\frac{1}{2}\sin(\frac{\pi }{12}\hat{t}+2.8)+21\;, \end{array}$$ (4)

where $\hat{t}$ denotes time, in hours since morning wake time (see blue and green curves in Fig 4B). The amplitudes of the daily modulation of response frequencies (±6 Hz for A*β*-fibers and ±0.5 Hz for C-fibers) were chosen to fit the model’s simulated pain signal, namely the firing rate of the projection neuron population, to the experimental measurements of pain sensitivity, as described below.

To model the effects of the A*β*-dependent presynaptic inhibition of C-fiber activity mediated through an additional inhibitory interneuron population (I_2_), we assume that the *I*_2_ population is only activated by high, stimulus-induced activity of the A*β*-fibers and that its activity tracks the daily modulation of ${\text{R}}_{\text{A}\beta }\left(\hat{t}\right)$ but at a lower firing rate. As a result, presynaptic inhibition lowers the stimulus response frequency of C-fiber activity, ${\text{R}}_{\text{C}}\left(\hat{t}\right)$, as follows:

$$\begin{array}{c} {\text{R}}_{{\text{C}}_{\text{eff}}}\left(\hat{t}\right)={\text{R}}_{\text{C}}\left(\hat{t}\right)-g_{\text{A}\beta \text{C}}\left({\text{R}}_{\text{A}\beta }\left(\hat{t}\right)-30\right)\;, \end{array}$$ (5)

where *g*_A*β*C_ scales the effects of the presumed *I*_2_ activity (see black dashed curve in Fig 4B), and the -30 mimics the lower *I*_2_ firing rate. This presumed level of *I*_2_ activity maintains effective C-fiber activity on the same scale as the original C-fiber activity, see blue solid and black dashed lines in Fig 4B.

**Fig 5:**
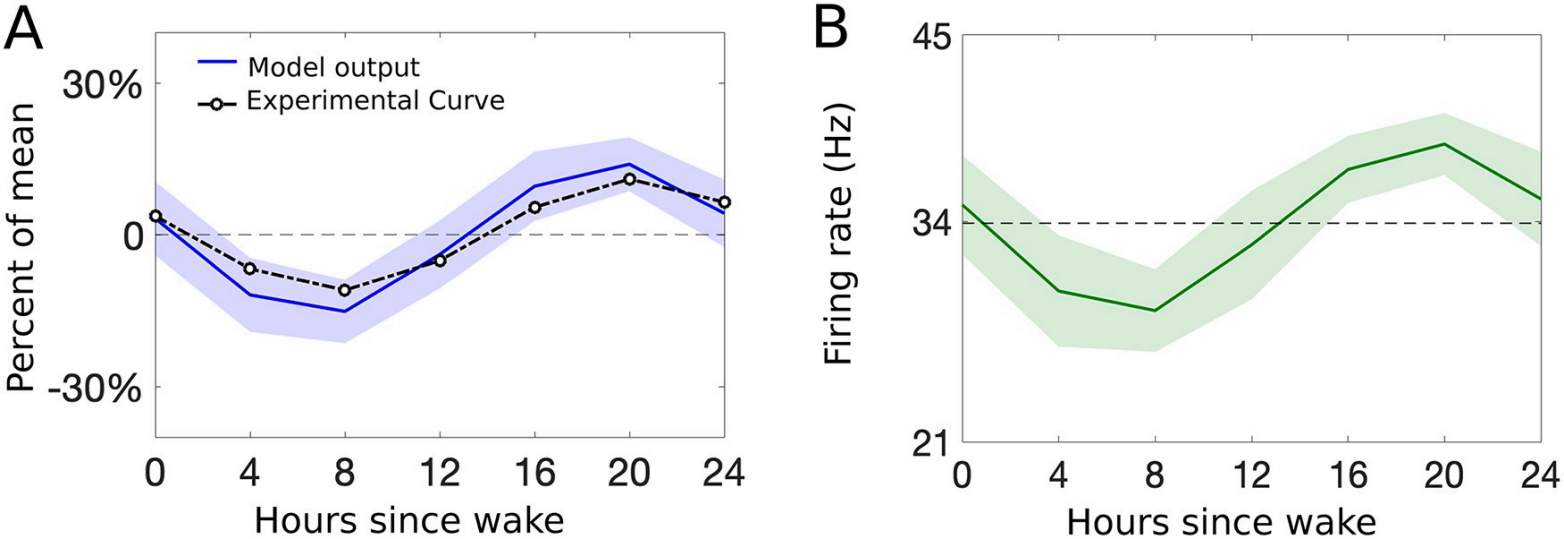
Daily rhythmicity of pain sensitivity: Comparing model to experiments. A: Percent of the mean response of the model output (blue) plotted as mean (curve) and standard deviation (shaded region) over 30 realizations of the Poisson input. The fitted curve from Fig 4A is plotted in black open circles. B: Firing rate of the P population in response to the C-fiber input as a function of the time of day. The x-axis refers to hours since the typical or scheduled morning wake time.

We note that while the daily modulation of the stimulus response frequencies governing spikes on the afferent fibers is on the order of hours, our model output changes on the order of fractions of seconds (e.g., *τ*_*P*_ = 0.001 s). Because of such a difference in time scales, there is only a small change in the stimulus frequencies ${\text{R}}_{\text{A}\beta }(\hat{t})$ and ${\text{R}}_{\text{C}}(\hat{t})$ during the response to a brief nociceptive stimulus. Hence, we consider specific time points at a constant $\hat{t}$ in a 24-hour period (see Fig 4B) when generating the (daily modulated) response of afferent fibers to stimulation. We compare the 24-hour rhythm in pain sensitivity computed by our model with the sinusoidal curve representing the human daily pain sensitivity fitted to experimental data in Fig 4A. Introducing the rhythmicity of fiber responses described above, we simulate our model equations at 7 time points over the 24-hour day, recording our model output (firing rate of the projection (P) neuron population) for each time point. To compare with the experimental curve, we compute variation as a percent of the mean by calculating the mean of the average response firing rates of the P population to stimuli given over the whole day, and comparing the firing rate at each time point during the day to that mean firing rate. Fig 5A shows the model pain sensitivity as a percent of the average over the day (blue curve) as compared to the experimental pain sensitivity (black dashed curve). Notice that the average firing rate of the P population, as shown in Fig 5B, is above 25 Hz which can be considered as a threshold for pain (see [44]). Furthermore, for the daily rhythmicity of pain sensitivity, the model output represented in terms of percent of mean (firing rate of the P population) closely follows experimental results (see Fig 5A).

## Results

### Model output

**Fig 6:**
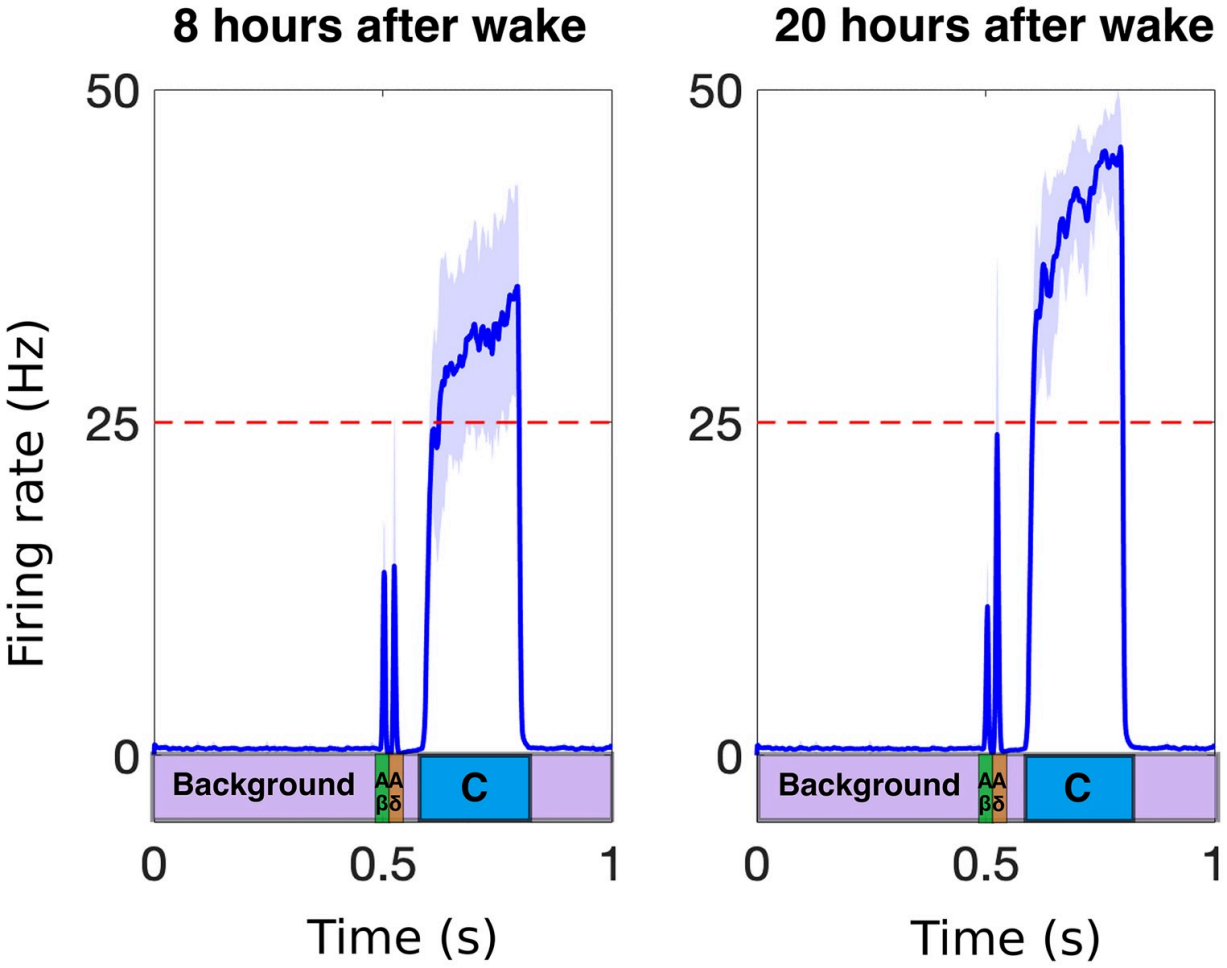
An example output of the PNs at two time points averaged over 30 realizations. The average firing rate of the P (projection neuron) population during (left) afternoon (lowest pain sensitivity) and (right) early morning (highest pain sensitivity) in response to a brief nociceptive stimulus and modulation in sensitivity of the afferent fibers over the day. The thick curve denotes the mean, the shaded region the standard deviation, of 30 realizations of the Poisson spiking activity on the afferent fibers (for one realization, see Fig 3). We interpret P firing rate frequencies higher than 25 Hz (dashed line) as painful.

To simulate response to a brief painful stimulus at the periphery, we construct average firing rate functions for activity of the A*β*-, A*δ*- and C-fibers based on the time of day as input to the DH, and calculate the resulting behavior of the PNs as described by the equations in (1). Fig 6 displays the average firing rate of the P population in response to nociceptive stimuli at two time points during the 24-hr day. Our model reproduces the average firing-rate pattern of the populations of neurons in the DH when the three afferent fibers differ in their conductance speeds, as noted by three distinct activations of the PNs in Fig 6. We follow [44], and interpret the painful response as the firing rate of the PNs crossing a threshold of 25 Hz. The average firing rate of the P population is qualitatively similar to that seen experimentally (e.g., see Fig 1a in [39]) and agrees with the daily variation in pain as reported in [13] (lower sensitivity in the afternoon and higher sensitivity at night).

Note here that we are only considering nociceptive stimulation of the afferent fibers as mechanical stimulation may follow a different circuit within the DH or more complicated activation of the different afferent fibers. To quantify the amount of pain experienced from the stimulation of the afferent fibers, we take the average firing rate of the PNs over the period of time when the C-fibers’ response has reached the DH (see blue rectangle in Fig 6). Note that the amount of time that the C-fiber response is activated is constant across the day and we consider the average firing rate above 25 Hz as painful [44].

The parameters for this model were chosen to give painful responses (i.e., firing rate of the P population above 25 Hz), but also to allow the neuron populations to reach their maximal firing rates during times of day with highest pain sensitivity. We note that the input from the spinal cord is only one component to the overall experience of pain. The P population reaching a maximum represents the maximum possible nociceptive response from this portion of the spinal cord. Thus, (and as concerns all of the simulations of our model) a maximal firing rate of the P population does not necessarily correspond to the maximal pain experience. Additionally, the chosen parameter set allows our model to sufficiently capture experimentally-observed phenomena such as wind-up and pain inhibition, but we recognize that this is not the only set of parameters that would yield these results. For a complete description of the parameter value choices, see S1 Table.

### Model validation: Wind-up

In addition to the example model output in Fig 6, we further validate our DH circuit model by showing that it reproduces *wind-up* —that is, increased (and frequency-dependent) excitability of the neurons in the spinal cord due to repetitive stimulation of afferent C-fibers [45]. Wind-up serves as an important tool for studying the role of the spinal cord in nociception and has often been used as an example phenomenon to validate single neuron models of the DH (see [21, 27, 28], for example). However, both the physiological meaning and the generation of wind-up remain unclear (see [46] for a review).

There are several possible molecular mechanisms proposed for the generation of wind-up [46]. Earlier work on single neuron models suggests that wind-up is generated by a combination of long-lasting responses to NMDA-receptor-mediated synaptic currents and membrane calcium currents providing for cumulative depolarization of the PNs [27]. Indeed, calcium conductances and NMDA receptors of the projection/deep dorsal horn neurons are included in all previous models of the DH circuit [21, 27, 28]. In addition, the study done in [28] emphasizes the effect (direct or via influencing the dependence of the deep dorsal horn neurons on their intrinsic calcium currents) NMDA and inhibitory synaptic conductances have on the extent of wind-up in the deep DH neurons [28].

**Fig 7:**
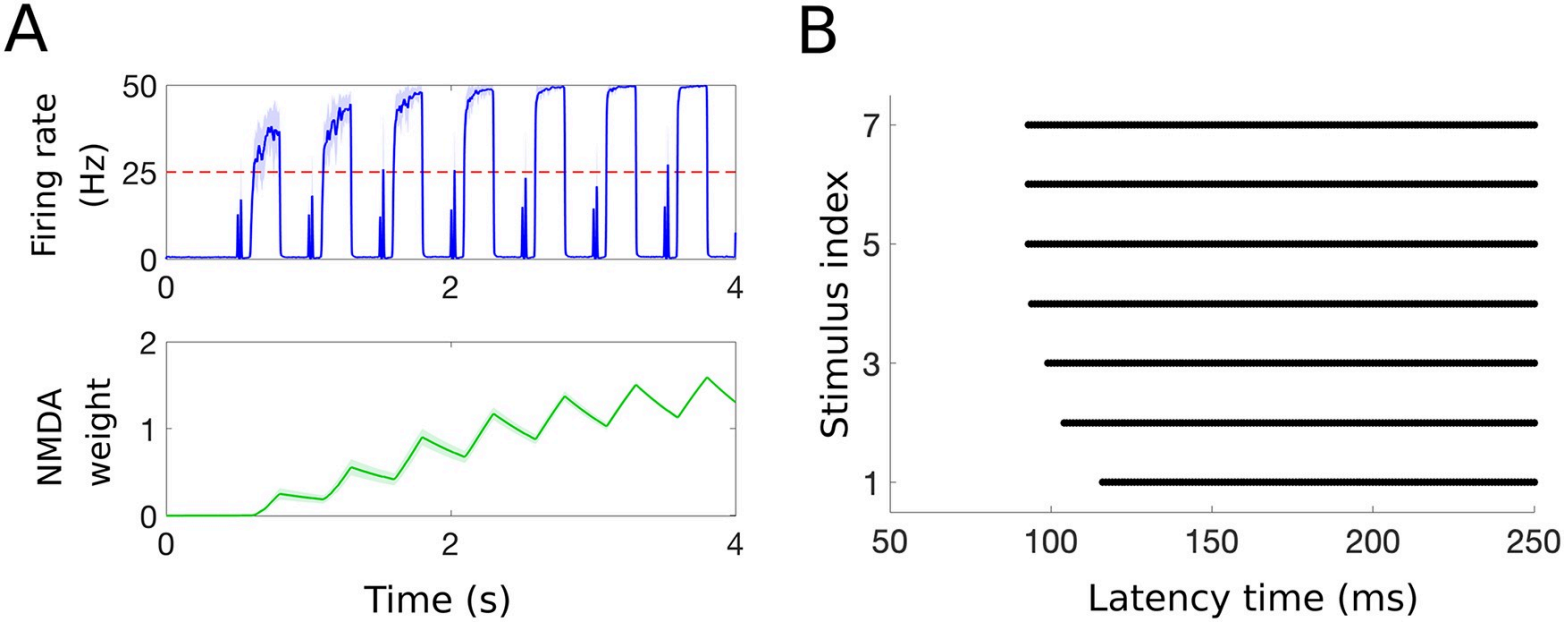
Modeling the wind-up phenomenon. A: Simulated average firing rate of the P population (top) and NMDA synaptic weight, *g*_NMDA_ (bottom), in response to a repeated brief nociceptive stimulus (at 2 Hz). In the top panel, the blue curve denotes the mean and the shaded region denotes the standard deviation in response to 20 realizations of the stimulus induced activity of afferent fibers. B: Pain latency computed for a repeated stimulus at 2 Hz. Latency is defined here as the first time when the average firing rate of the P population exceeds the threshold of 25 Hz (interpreted as painful) and decreases as a function of the stimulus index (solid lines indicate times when average firing rate of P exceeds painful threshold). The latency dynamics match those found in experiments [47].

As noted in the Methods section, we incorporate NMDA synapses into our model for the DH circuit by taking into account that the dynamics of the synaptic weight of the connection from the C-fibers to the PNs, *g*_NMDA_, depends on the average firing rate of the P neuron population [see Eq (2)]. We assume that the dynamics of *g*_NMDA_ are much slower than those of the neuron populations (*τ*_NMDA_ = 1 s while, e.g., *τ*_P_ = 0.001 s). As a result, in response to a repeated stimulus (i.e., when the model input as shown in Fig 3 is presented to the DH circuit at a frequency of 2 Hz), the average firing rate of the P population during the C-response increases (see top panel in Fig 7A) and the synaptic weight *g*_NMDA_ exhibits slowly increasing dynamics in response to the increased activity in the P population (see bottom panel in Fig 7A). For a repeated stimulus at 2 Hz, the latency, which we consider as the time from the start of the stimulus (*t* = 0.5 s) to the time when the average firing rate of P exceeds 25 Hz (i.e., considered as painful), decreases with the stimulus index (i.e., index 1 denotes the first stimulus in the repeated sequence), see Fig 7B, as seen in experiments [47].

**Fig 8:**
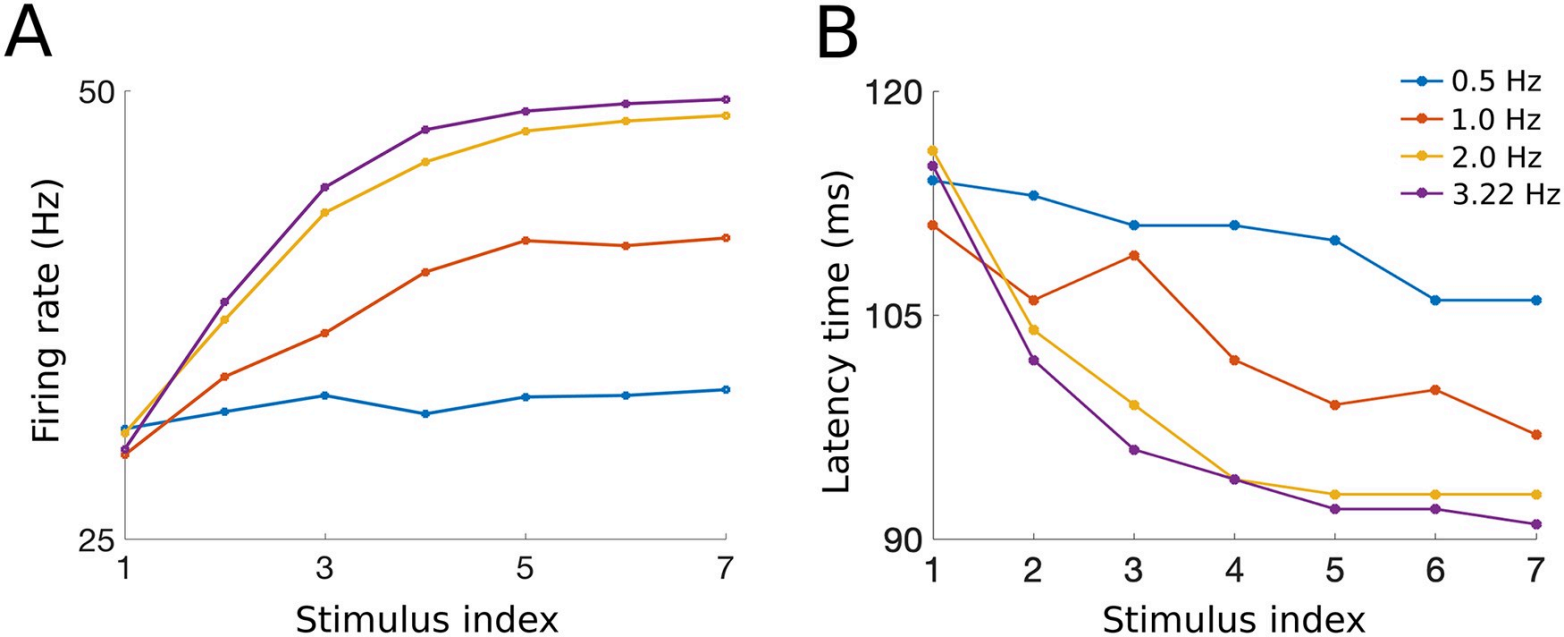
Characterizing how wind-up phenomenon changes with frequency. A: The mean of the average firing rate of P neurons during the C-response (which we define as the time interval 90-300 ms after the start of the stimulus, see blue box on bottom of Fig 6) for each stimulus index increases as a function of the stimulus frequency. B: Latency, defined here as the first time when the average firing rate of P population exceeds the threshold of 25 Hz (interpreted as painful), decreases with the change in the frequency of the repeated stimulus.

However, the increase in the average firing rate of the P population depends on the frequency of the repeated stimulation, with optimal effects seen experimentally at stimulation frequencies between 1-3 Hz [46]. Our model captures the phenomenon of wind-up, as well as the frequency dependency. For example, when the model input is repeated at a frequency of 2 Hz, the mean of the average firing rate of the P population during the C-response (see blue box on bottom of Fig 6) increases from about 25 Hz during the first stimulus to about 50 Hz during the fifth stimulus similar to previous modeling results [21], while in the case of a stimulus repeated at 0.5 Hz, the mean P firing rate during the C-response does not change as a function of the stimulus index (Fig 8A, yellow curve vs blue curve). We note that we simulate frequencies up to 3.22 Hz as this is the highest frequency we can model without an overlap in the *P* neuron responses (see Fig 7A, top). We include it here to show the general trend of wind-up in response to an increase in frequency. It’s clear to see that as the frequency increases, and the responses are allowed to interact, the result would be a yet faster rise in the firing rate to its maximum due to the additive nature of the NMDA weight (see Fig 7A, bottom). We also show that the latency time decreases with increasing frequency, (see Fig 8B), with maximal effects seen for stimulation frequencies of 2-3 Hz and minimal effects seen for 0.5 Hz, as observed experimentally.

### Model application: Daily rhythm in the modulation of pain inhibition

**Fig 9:**
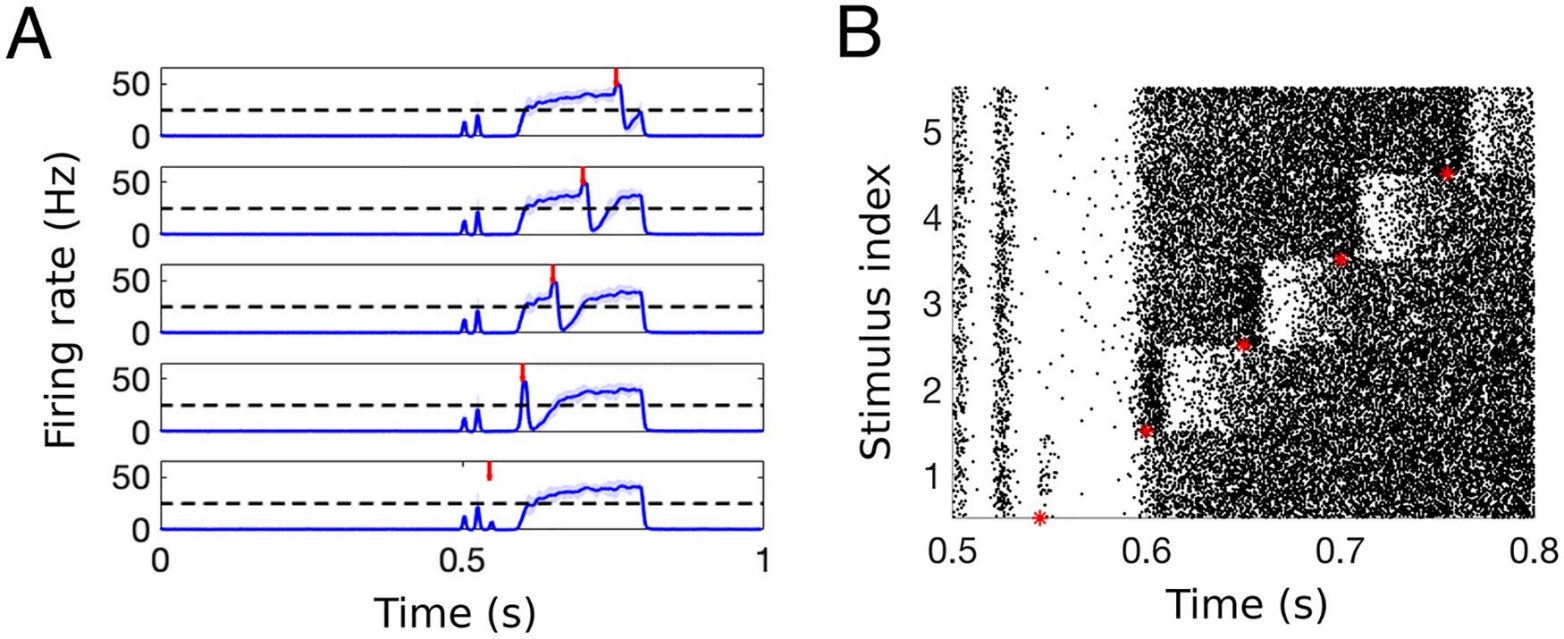
Inhibition of painful response by subsequent activation of A*β*-fiber activity. A: Firing rate of the projection neurons in response to secondary *Aβ* stimulation following brief nociceptive stimulus activating all three fibers. B: Spike raster plot generated from the model output shown in A for 1000 PNs constructed to resemble Fig 5 in [21], which replicates experiments from [47]. We denote the arrival of the second A*β*-pulse with a red tick mark (asterisk) in A (B).

It has been experimentally observed that stimulation of A-fiber afferents can lead to inhibition of the activity of the PNs that typically follows from stimulation of C-fiber afferents [21]. This is related to the idea that when you stub your toe, you immediately apply pressure on the toe and feel some lessening of pain. To capture this phenomenon in our model, we simulate a brief painful stimulus at the periphery that activates all three fibers (stubbing of the toe) and then deliver a second brief stimulation to the A*β*-fibers a short time thereafter (pressure applied to toe), shown in Fig 9 by the red arrows. The arrival time of the second pulse to the A*β*-fibers is increased by 50 ms in each simulation, and the response in the projection neurons is shown in blue. For comparison with experimental data in [47] and model simulations in [21], we visualize the average firing rates of P predicted by our model (Fig 9A) with a spike raster plot in Fig 9B. That is, we derive firing times from the numerically computed average firing rates of the P population, as explained in [48]. As the timing of the second pulse gets closer to the arrival of the C-fiber stimulation at the DH, there is a brief period of excitation followed by a longer period of inhibition, as seen in experiments [47].

**Fig 10:**
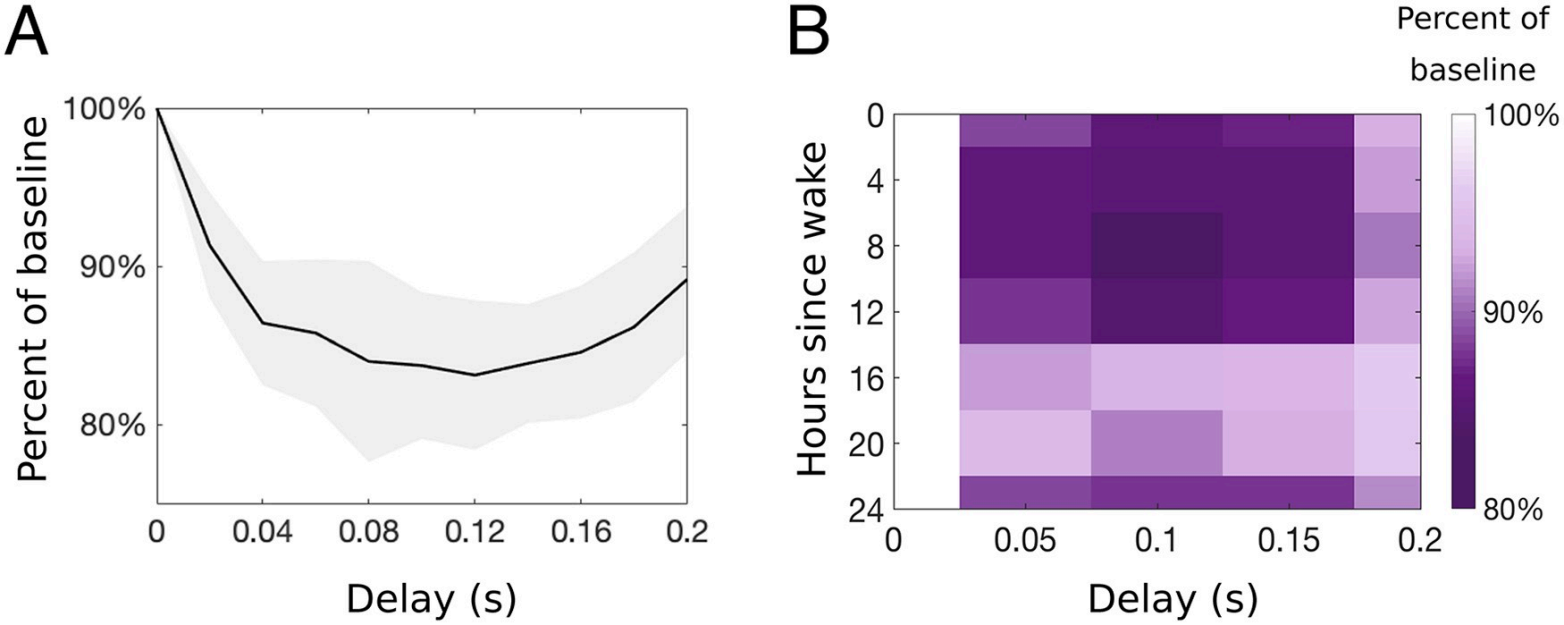
Quantifying effectiveness of pain inhibition across the day. A: Percentage of the baseline firing rate of the PNs as a function of the delay time of the second *Aβ* pulse at 8 hours following the typical morning wake time. B: Percentage of the painful response as a function of both time of day and delay time of the second A*β* pulse. Color scale indicates the percent of the baseline firing rate of the PNs with no pain inhibition (i.e., 0 s delay), with darker colors representing larger decreases in pain as a result of the pain inhibition.

While only qualitative descriptions of pain inhibition are reported in [47], we quantify the amount the painful response is suppressed by the second activation of the A*β*-fibers by comparing the average firing rate of the P population during the C-response in each panel of Fig 9A (thick curves) to that in the bottom panel in Fig 9A where the secondary A*β* activation has no effect on the C-fiber response (defining a baseline firing rate). The percent of this baseline firing rate is plotted in Fig 10A as a function of the delay time of the second A*β* pulse (relative to the time of the original nociceptive stimulus). Note that the pain response decreases as the delay of the second A*β* pulse increases and its arrival time coincides with the C-response, as reported in [47].

We use our model of pain sensitivity to investigate the daily rhythmic effects on the phenomenon of pain inhibition. Fig 10B demonstrates changes in the percent of baseline firing rate of the P population during the C-response as a function of the delay in the second A*β* pulse, for each time of day (i.e., in hours since morning wake time). Our model predicts that pain inhibition is most effective during early afternoon (4-8 hours after wake), when the A*β*-fibers are the most sensitive to external stimulus (i.e., their stimulation frequency is at its highest daily value) and the C-fibers are the least sensitive to external stimulation. This can be seen in the color plot in Fig 10B by the dark horizontal band around 4-8 hours after wake (middle of the afternoon) for all delays. Notice that for 16-20 hours after wake (middle of the night), the pain percentage is very high and there is little change in the percent of pain as a function of delay time, indicating that pain inhibition is not very effective at these times.

In addition to predicting the time of day that pain inhibition is most effective (mid-afternoon), our model also predicts that a delay from 0.05 to 0.15 seconds after the original painful stimulus is ideal for the optimal lessening of pain experienced, as can be seen in both plots of Fig 10 by these particular delay times showing the lowest percent of pain response for all times of day.

### Model prediction: Neuropathy

Neuropathic pain occurs due to various conditions involving the brain, spinal cord, and nerve fibers. It is distinguished from inflammatory conditions like arthritis in that it often appears in body parts that are otherwise normal under inspection and imaging, and is also characterized by pain being evoked by a light touch. Experiments on pain sensitivity in neuropathic patients suggest that neuropathic pain has a daily rhythm as well [15, 49–52], having its peak in the afternoon [53]. An afternoon peak in pain sensitivity is the reverse of the daily rhythm in pain sensitivity under normal conditions [6]. Nerve injury can cause a dysregulation of chloride ion transporters that control intracellular chloride concentration in DH neurons (reviewed in [54]). Maintenance of a low intracellular chloride concentration is important for the functioning of inhibitory neurotransmission. Under typical conditions, the binding of the neurotransmitter GABA on postsynaptic receptors produces an inhibition of postsynaptic activity by allowing negatively-charged chloride ions to flow into the postsynaptic neuron, thus producing hyperpolarization (or decrease in membrane voltage). If intracellular chloride concentrations stay semi-permanently elevated, chloride ions may flow out of the cell in response to GABA receptor activity producing excitatory rather than inhibitory effects. Several authors have hypothesized that dysregulation of inhibition in spinal pain processing circuits could explain the development of pain sensation in response to non-noxious stimuli under neuropathic conditions [54, 55]. Specifically, several authors [54, 56, 57] implicated a switch in presynaptic inhibition to presynaptic excitation in the DH as one culprit for eliciting neuropathic pain phenomena.

As a result, we set out to determine if a switch from presynaptic inhibition to presynaptic excitation in our model is sufficient to replicate the experimentally-observed 8-12 hour change in the phasing of daily rhythms in pain sensitivity under neuropathic conditions. We show that our model can capture such an inversion of the rhythmicity of the firing rate of the PNs with a change from inhibition (normal conditions) to excitation (neuropathic conditions) in the presynaptic influence of the A*β*-fibers on C-fiber synaptic signaling. The location where the change from inhibition to excitation occurs is denoted in our model diagram by the two asterisks in Fig 1. Thus, we assume that under neuropathic conditions, the connection from I_2_ to the synaptic terminals of E and P is excitatory instead of inhibitory.

**Fig 11:**
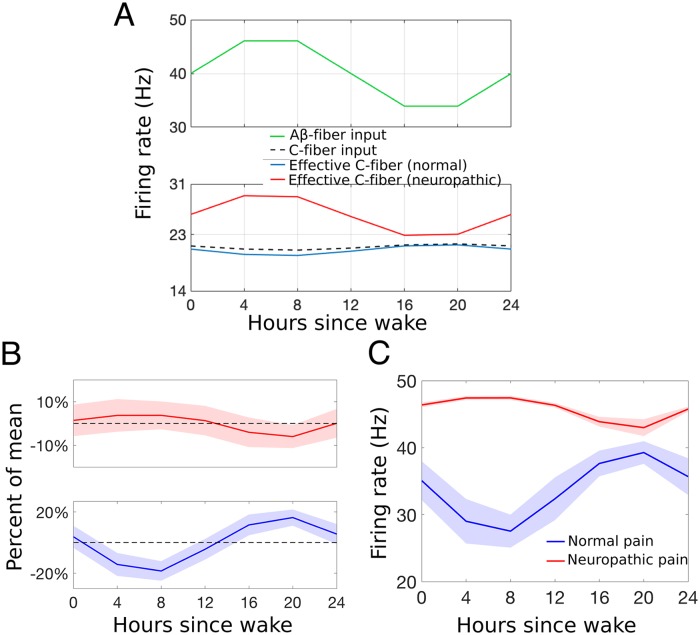
A change in the daily rhythms of pain sensitivity in neuropathic pain conditions compared to normal conditions. A: Daily variation of the stimulus response firing rates of the A*β*- (top panel) and C-fibers (lower panel, dashed curve), and effective C-fiber stimulus response firing rate including effects of A*β*-dependent presynaptic inhibition under normal conditions (lower panel, blue curve, same as in Fig 4B) and A*β*-dependent presynaptic excitation under neuropathic (red curve) conditions. B, C: Daily variation in the response of the PNs to a brief nociceptive stimulus quantified by the percent of its mean (B) and average firing rate of the C-fiber response (C) for normal (blue, same as in Fig 5) and neuropathic (red) conditions. The x-axis refers to hours since the typical or scheduled morning wake time.

Recall from the Methods section that we model presynaptic inhibition as an A*β*-dependent decrease in the stimulus response firing rate of the C-fibers [see Eq (5)]. The assumption that presynaptic inhibition turns to excitation results instead in an A*β*-dependent increase in the C-fiber stimulus response firing rate represented by the following equation

$$\begin{array}{c} R_{C_{\text{eff}}^{\text{neuro}}}\left(\hat{t}\right)=R_{C}\left(\hat{t}\right)+g_{\text{A}\beta \text{C}}^{\text{neuro}}\left(R_{\text{A}\beta }\left(\hat{t}\right)-30\right), \end{array}$$ (6)

where $g_{\text{A}\beta \text{C}}^{\text{neuro}}$ is the strength of the effect of A*β*-fiber activity on C-fiber activity under neuropathic conditions (see red curve in lower panel of Fig 11A). This daily variation in the stimulus response frequency of C-fiber activity results in the desired inversion of projection neuron population firing rate response to a brief nociceptive stimulus (Fig 11C), and thus pain sensitivity (Fig 11B), across the day. Thus, under normal conditions, the pain sensitivity rhythm follows the daily rhythm of the C-fibers (compare blue curves in all panels) but mimics the rhythm in the A*β*-fibers under neuropathic conditions (compare red curves in B and C with green curve in A).

In our model, we obtain this inversion of rhythm in pain sensitivity by assuming that A*β*-dependent presynaptic excitation under neuropathic conditions has a larger magnitude than presynaptic inhibition under normal conditions. Specifically, the weighting factor $g_{A\beta C}^{\text{neuro}}=0.25$ under neuropathic conditions is larger than *g*_*AβC*_ = 0.05 under normal conditions. This can be interpreted as an increase in firing rates of the A*β*-fibers under neuropathic conditions that results in increased excitation of the I_2_ inhibitory population, and thus larger magnitude of presynaptic excitation compared to presynaptic inhibition under normal conditions. There are several proposed mechanisms for the many types of neuropathic pain, some of which show increased activity of the A*β*-fibers [41].

**Fig 12:**
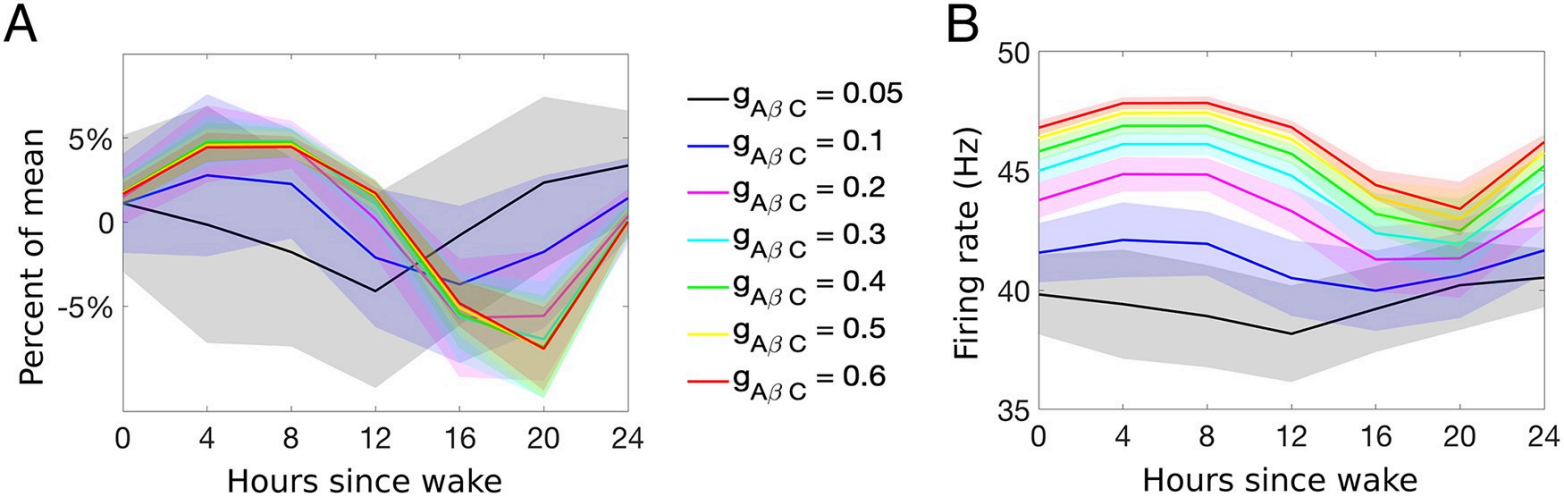
Investigating the weighting parameter, $g_{A\beta C}^{\text{neuro}}$. A: Percent of the mean and B: average firing rate of the PNs in response to changes in the strength of excitation, during neuropathy, from the A*β*- fibers to the C-fibers, $g_{A\beta C}^{\text{neuro}}$ (denoted by *g*_*AβC*_ in the figure). The x-axis refers to hours since the typical or scheduled morning wake time.

To investigate the dependence of the magnitude of A*β*-dependent presynaptic excitation on the inverted daily rhythm, we simulate the model response to brief nociceptive stimuli across the day for different values of the weighting parameter $g_{A\beta C}^{\text{neuro}}$ (see Fig 12). Results show that weak presynaptic excitation (black and blue curves) reduces the amplitude of daily variation in P population firing rates and does not induce an inverted rhythm. For larger values of $g_{A\beta C}^{\text{neuro}}$, the correct rhythmicity is obtained and amplitude increases but eventually saturates. Larger magnitudes of presynaptic excitation only serve to increase the firing rate over the entire day, thus increasing the average over the entire day and not affecting the variation in percent of the mean.

Note that the amplitude of the rhythmicity of pain sensitivity under neuropathic conditions is small, about 5% as compared to 15% under normal conditions. There are few experimental studies that measure the amplitude of modulation of pain sensitivity under neuropathic conditions; however, one study shows neuropathic pain sensitivity to have a similar amplitude to, if not slightly larger than, acute pain [51]. Our model proposes that the rhythm is intrinsic to the afferent fibers, however many believe that there may also be daily rhythms within the top-down inhibitory modulation of many of the neuronal populations in the pain-processing circuit [58]. With this initial hypothesis of rhythmicity in the fiber input, our model replicates the overall increase in pain sensitivity under neuropathic conditions, reflected by increased firing rates of the PNs. Indeed, our model simulations suggest that inhibition turned excitation at the level of the fibers is a possible mechanistic explanation for the inversion of pain sensitivity rhythms seen under neuropathic conditions. Modulation by daily rhythms could also be explored in alternative parts of the pain processing circuit, including the output of the projection neurons and its propagation along the spinal cord. These additional mechanisms in combination with disinhibition may enhance the modulation of the daily rhythm of pain sensitivity under neuropathic conditions.

## Discussion

We have developed a firing-rate model for the processing of nociceptive stimuli in the DH of the spinal cord, with a particular interest in investigating the daily rhythmicity of pain sensitivity. Our model follows the formalism of many neuron firing-rate-based models, but to our knowledge, it is novel for pain processing in the spinal cord. In addition to accounting for typical pain phenomena such as wind-up and pain inhibition, our model captures the rhythmicity in pain sensitivity over the 24-hour day mediated by intrinsic rhythmicity of afferent fiber activity. We include experimentally-justified presynaptic inhibition from the A*β*-fibers to the C-fibers, and show how disinhibition of this pathway under neuropathic conditions is sufficient to induce the experimentally-observed inversion of the rhythmicity of pain sensitivity. Our minimalistic model is based on physiology and thus provides an accessible theoretical framework for experimental and clinical investigations of diverse physiological processes modulating pain processing in humans.

In contrast to a detailed biophysical model of a single neuron [21, 27, 28] or a large-scale network of individual neurons [44], we construct equations to describe the population activity of projection, inhibitory, and excitatory neurons in the DH. As a result, we work with average firing rates for each of the three neuron populations according to the formalism developed in [29]. Therefore, our modeling approach is similar to [26] but our model predictions are in terms of average firing rates of neuron populations instead of potentials of individual cells. In our choice of model formalism, we assume that the neurons in each population behave similarly, i.e., they receive similar inputs and respond similarly to those inputs, such that we can consider the average behavior over all neurons in each population as the primary mode of information transfer in the circuit. This is a limitation in the sense that often interesting phenomena in neuroscience arises from the nonlinear interactions between neuron spike timings and their differences in interpreting incoming stimuli. However, results from other modeling approaches that replicate spiking behavior [21, 44] have not indicated that discrimination of spike timings contributes substantially to spinal pain processing. Additionally, some parameters in this model formalism cannot be easily obtained from experiments. For example, the weights with which one population influences another [see *g*_PrePost_ in Eq (1)], represent an average synaptic strength from all neurons in one population to all neurons in another, which cannot be measured experimentally. We choose parameter values for these weights in order to replicate experimental data on the response of the PNs under different conditions. Finally, although there is experimental evidence to show that an increase in the activity of the PNs correlates with an increase in pain sensation [32], the choice of instituting a threshold of 25 Hz on the PNs above which the model output is considered painful is somewhat arbitrary. However, we follow this convention used in [44] because to our knowledge a more physiologically accurate approximation has not yet been determined.

The circuitry of our DH model is based on the gate control theory of pain [22, 24], similar to previous mathematical models for spinal nociception processing [21, 26–28]. While using different model formalisms, circuit activity in these models centers around inhibition of PN responses to C-fiber input by A*β*-fiber activity. In this way, A*β*-fiber activity gates responses to nociceptive stimuli. More recent results have called into question gate control theory [25]. In particular, a large-scale network model of spinal cord neural circuitry has been constructed [44] that includes numerous known cell types, their laminar distribution, and their modes of connectivity. This model has been used to investigate the mechanisms of pain relief through dorsal column stimulation (DCS), a procedure to treat neuropathic pain. The results shown in [44] identify limitations of the gate control theory and propose alternate circuitry that more accurately accounts for the effects of DCS on nociceptive and neuropathic pain.

As concerns our model predictions for neuropathy, the low amplitude of the neuropathic pain rhythm in the model output may suggest that a simple spinal cord model is not sufficient to completely describe the phenomenon of an inversion in the rhythm of pain modulation under neuropathic vs normal conditions. Indeed, the daily rhythm that we use in the model is likely to reflect both the influences of circadian rhythms and sleep homeostasis, of which the sleep homeostatic component presumably increases throughout the evening, and therefore, would potentially amplify the peak in the neuropathic pain rhythm that occurs during that time. Furthermore, our current model does not include top-down modulation of spinal pain processing from the brain for which there is experimental evidence in support of circadian regulation of top-down inhibition [5, 59].

In this study, we do not consider the neuropathic property in which patients experience pain in response to a non-noxious, mechanical stimuli. Instead, we restrict our attention to the response to nociceptive stimuli since mechanical stimulation may be processed by different pathways. Nonetheless, our model predicts that neuropathic conditions can, in part, be explained by A*β*-dependent presynaptic excitation of C-fiber synaptic signaling that is of a larger magnitude than the presynaptic inhibition that occurs under normal conditions. Specifically, to obtain the experimentally-observed inversion in the rhythmicity of pain sensitivity experienced by neuropathic patients, our model predicted an increase in the A*β*-dependence on C-fiber stimulus response [compare $g_{\text{A}\beta \text{C}}^{\text{neuro}}$ in Eq (6) to *g*_A*β*C_ in Eq (5)]. This increase could potentially be due to increased response firing rates of A*β*-fibers, as well as by increased efficacy of the excitatory effects of the secondary inhibitory population *I*_2_. These effects cause an increase in firing rates of PNs in response to brief nociceptive stimuli, but could also contribute to increases in PN responses in mechanical stimuli processing pathways. Additional studies on the interaction of the pathways processing non-noxious and nociceptive stimuli, and their properties under neuropathic conditions are needed to fully understand this phenomenon.

Often it is difficult, if not impossible, to experimentally measure properties of individual neurons in vivo, and in response to all possible nociceptive (and mechanical) stimuli. Due to this lack of knowledge, it is often impractical to build detailed models of DH neurons in which many parameters would need to be determined from biological data. In this respect, simpler population firing-rate models, like the one presented here, have an advantage in that there are significantly fewer parameters and they are constrained by measurements of more accessible macroscopic properties of the circuit. We have developed a novel firing-rate model for the neural circuit in the DH that processes nociceptive stimuli and we have shown that it can capture the same experimentally-observed phenomena as more detailed models. Additionally, we were able to clearly propose and test a mechanism for the daily rhythm in pain sensitivity and modulations of that rhythmicity under neuropathic conditions. Given its accessibility compared to more detailed or larger biophysically-based models, our model is suitable for including experimental results, e.g., on the activity of the afferent fibers, and appropriate for experimental and clinical investigations of diverse physiological influences on pain processing, such as the effects of sleep deprivation on pain sensitivity [60] or the mechanisms underlying the efficacy of spinal cord stimulation for treatment of chronic pain conditions.

## Supporting information

S1 Text Detailed description of model parameter choices. (PDF)

S1 Table Summary of model parameters and their default values. (PDF)
