# Supplementary material for: Modeling the daily rhythm of human pain processing in the dorsal horn
Source: PLoS Comput Biol. 2019 Jul 11;15(7):e1007106. doi: 10.1371/journal.pcbi.1007106 (PMC6622484; doi:10.1371/journal.pcbi.1007106)
Supplement: S1 Text — (PDF) [file pcbi.1007106.s001.pdf]

Supporting Information

Jennifer Crodelle [1,◕], Sofia H. Piltz [2,◕], Megan Hastings Hagenauer [3], Victoria Booth [2],

1 Courant Institute of Mathematical Sciences, New York University, New York, NY 10012, USA, **2** Department of Mathematics, University of Michigan, Ann Arbor, Michigan, 48109, USA, **3** Molecular and Behavioral Neuroscience Institute, University of Michigan, Ann Arbor, Michigan, 48109, USA

◕These authors are joint first authors on this work.

* crodelle@cims.nyu.edu

For a list of all of the model parameters and their default values that we use in the simulations of our model [see Eq (1)], see S1 Table. (By the NMDA receptors in Table 1, we refer to those of the projection neuron population P.) We note that the values for the weight parameters (g_{PrePost}) are not a unique set of weights but a result of modeling choices as concerns the firing rates of the afferent fibers during baseline and stimulus (see Section 1.2). We base these choices on experimental data reported in [2, 5]. Similarly, we choose the parameters representing the sensitivity, half-activation threshold, and the maximum of the response functions (i.e., α, β, and max, respectively), as well as the intrinsic time scales (τ) of the neuronal populations and the NMDA receptors, in order to mimic experimental results as reported, e.g., in [4].

The intrinsic time constants for the E and I populations are chosen to mimic the synaptic time constants used in previous studies, see Table 1 in [4]. The time constant for the P population is chosen to be significantly faster than both E and I such that the response of the P population reflects the inputs from each individual fiber group, as was seen experimentally in [2, 3] and our model replicated in Figure 6. The time scale for NMDA was chosen from [1], and lengthened to replicate the frequency effects of wind up, see Figure 8.

As concerns the weights g_{PrePost} scaling interactions between the populations, many of these parameters are interdependent and the effect of changing just one is not immediately obvious. We will explain, to the best of our ability, the reasoning behind

each choice and which parameters are more sensitive than others. First, notice that in Figure 12 we perform a sensitivity analysis on the weight from the I_2 interneuron population to the P population. This is an example of a parameter that, with all other weights held constant, the effect on the firing of the P population can be explicitly shown. Other weights are more interdependent. For example, consider the weight from the Aβ-fibers to the I population, $g_{A\beta I}$. Changes in this weight will increase the firing rate of the I population, and thus allow more inhibition onto the P population. This effect can be mediated, and even cancelled out, by simply decreasing the weight from the I population to the P population, g_{IP}.

One of the constraints used in determining the values of the weights are that each population must have a nonzero firing rate when the fibers are activated; in particular, we require that the P neurons have a firing rate close to the painful threshold of 25 Hz when the C-fibers are activated (i.e., that we are indeed eliciting a painful response). This in itself leads to a range of values for the weights from the incoming fibers to each population, $g_{A\delta P}, g_{A\beta P}, g_{A\beta I}, g_{CE}$. Then, we must choose the weights between the populations such that we get qualitatively similar responses for each phenomenon. For pain inhibition, the weights along the pathway from the $A\beta$-fibers to the I population to the P population, $g_{A\beta I}, g_{IP}$ are narrowed down. And for wind-up, the weights along the pathway from the C-fibers to the E population and from the E population to the P population, g_{CE} and g_{EP}, and along the pathway from the C-fibers to the P population, g_{CP}, are narrowed down to qualitatively capture wind-up and its frequency effects. Finally, all of these weights must work together to ensure that the firing rate of the P population in response to C-fiber activation is above 25 Hz and follows the experimentally-observed modulation of its percent mean across the day. The main objective is to find a set of weights that captures all three experimental phenomena: pain inhibition, wind-up, and daily modulation of pain sensitivity. Then, keeping all weights fixed, we apply the model to the phenomenon of neuropathy and investigate a mechanism underlying the inversion of the daily rhythm in pain sensitivity.

We note that due to this dependence of one weight on another, it is difficult to specify all possible combinations of weights that will yield similar results. We emphasize that these weights have limited biophysical interpretation and thus cannot be experimentally measured. We state that small changes to the weights will not greatly

affect these results and that we make no substantive claim that the values for these weights reflect actual synaptic currents. Instead, we focus on the conclusion that by finding a set of weights that can produce a model that qualitatively matches multiple experimental measurements of activity and phenomena in the spinal cord, we can use it to make predictions about the rhythmic activity of the neurons in DH under neuropathic conditions.

References

1. Aguiar P, Sousa M, Lima D. NMDA channels together with L-type calcium currents and calcium-activated nonspecific cationic currents are sufficient to generate windup in WDR neurons. J Neurophysiol. 2010;104(2):1155–1166.

2. Sikandar S, Ronga I, Iannetti GD, Dickenson AH. Neural coding of nociceptive stimuli-from rat spinal neurones to human perception. Pain. 2013;154(8):1263–1273. doi:10.1016/j.pain.2013.03.041.

3. Reeve AJ, Walker K, Urban L, Fox A. Excitatory effects of galanin in the spinal cord of intact, anaesthetized rats. Neurosci Lett. 2000;295(1-2):25–28.

4. Zhang TC, Janik JJ, Grill WM. Modeling effects of spinal cord stimulation on wide-dynamic range dorsal horn neurons: influence of stimulation frequency and GABAergic inhibition. J Neurophysiol. 2014;112(3):552–567.

5. Koltzenburg M, Handwerker HO. Differential ability of human cutaneous nociceptors to signal mechanical pain and to produce vasodilatation. Journal of Neuroscience. 1994;14(3):1756–1765.
