# Supplementary material for: Modeling the daily rhythm of human pain processing in the dorsal horn
Source: PLoS Comput Biol. 2019 Jul 11;15(7):e1007106. doi: 10.1371/journal.pcbi.1007106 (PMC6622484; doi:10.1371/journal.pcbi.1007106)
Supplement: S1 Table — (PDF) [file pcbi.1007106.s002.pdf]

Name	Description	Value	Source
	From literature		[2, 4]
$\max_{\mathrm{P}}$	maximum firing rate of neuronal population P	50 Hz	
$\max_{\mathrm{E}}$	maximum firing rate of neuronal population E	50 Hz	
$\max_{\mathrm{I}}$	maximum firing rate of neuronal population I	80 Hz	
	Fitted to experimental data, see Figure 2		[4]
α_{E}	sensitivity of the response in the E population	5.2	
α_{I}	sensitivity of the response in the I population	9.5	
α_{NMDA}	sensitivity of the response in the NMDA receptors	10.0	
α_{P}	sensitivity of the response in the P population	11.5	
β_{E}	half-activation threshold of the E population	29.2	
β_{I}	half-activation threshold of the I population	28.0	
β_{NMDA}	half-activation threshold of the NMDA receptors	38.0	
β_{P}	half-activation threshold of the P population	28.2	
	From literature		[1,4]
τ_{E}	intrinsic time scale of neuronal population E	10 ms	
τ_{I}	intrinsic time scale of neuronal population I	20 ms	
τ_{NMDA}	intrinsic time scale of the NMDA receptors	1 s	
τ_{P}	intrinsic time scale of neuronal population P	1 ms	[3]
	Chosen to replicate experimental responses to nociceptive stimuli		
$g_{A\beta\mathrm{C}}$	weight of the presynaptic inhibition from Aβ-fibers to C-fibers	0.05	
$g_{A\beta\mathrm{C}}^{\mathrm{neuro}}$	neuropathic weight from Aβ-fibers to C-fibers	0.25	
$g_{A\beta\mathrm{I}}$	synaptic weight from Aβ-fibers to neuronal population I	0.8	
$g_{A\beta\mathrm{P}}$	synaptic weight from Aβ-fibers to neuronal population P	0.8	
g_{CE}	synaptic weight from C-fibers to neuronal population E	1.6	
g_{CP}	synaptic weight from C-fibers to neuronal population P	0.8	
$g_{A\delta\mathrm{P}}$	synaptic weight from Aδ-fibers to neuronal population P	1.9	
g_{EP}	synaptic weight from E to P neuronal populations	0.35	
g_{IE}	synaptic weight from I to E neuronal populations	0.6	
g_{IP}	synaptic weight connection from I to P neuronal populations	1.8	
$\max_{\mathrm{NMDA}}$	maximum synaptic strength of the NMDA pathway	2.0	

Table 1: Summary of model parameters and their default values. The parameter values are either chosen from an experimentally determined range in the literature or they are chosen to replicate several experimental responses to nociceptive stimuli (i.e., pain inhibition, wind-up, etc.) discussed in this work.
